# Evaluation of owners’ and veterinarians’ attitudes toward veterinarian dressing styles in a teaching hospital in Taiwan

**DOI:** 10.1038/s41598-025-95715-2

**Published:** 2025-04-02

**Authors:** Kendy Tzu-yun Teng, You-Jing Shiu, Shang-Lin Wang

**Affiliations:** 1 Department of Veterinary Medicine, College of Veterinary Medicine, National Chung Hsing University, Taichung, 40227 Taiwan; 2 The iEGG and Animal Biotechnology Research Center, National Chung Hsing University, Taichung, 40227 Taiwan; 3 Department and Graduate Institute of Veterinary Medicine, School of Veterinary Medicine, National Taiwan University, Taipei, 10617 Taiwan; 4 Graduate Institute of Veterinary Clinical Sciences, School of Veterinary Medicine, National Taiwan University, Taipei, 10617 Taiwan; 5 National Taiwan University Veterinary Hospital, College of Bioresources and Agriculture, National Taiwan University, Taipei, 10672 Taiwan

**Keywords:** Accessory, Attire, Hairstyle, Impression, Veterinarian, Psychology, Health occupations

## Abstract

This study examined preferences for veterinarian dressing styles from both pet owners’ and veterinarians’ perspectives. A questionnaire was distributed to investigate the attitudes of pet owners and veterinarians toward six different dressing styles, four hairstyles, and nine appearance-related subjects toward both male and female veterinarians. A total of 211 pet owners and 92 veterinarians were included. Our results indicated that veterinarian dressing style affected the first impression of pet owners. Pet owners considered wearing a white coat or surgical scrubs an appropriate dressing style for veterinarians. While owners had no sex preference for veterinarians (*p* < 0.001), they had different attitudes towards various hairstyle for female and male veterinarians. Although any hairstyle, except for a bald hairstyle, was regarded as appropriate for female veterinarians among owners, male veterinarians with short hairstyles were considered more appropriate than other hairstyles. Wearing glasses, a name tag, a stethoscope, a watch, or sneakers tended to leave a neutral or appropriate impression. Wearing an earring, necklace, ring, or tattoo was considered very inappropriate by some owners. Overall, the owners were stricter than the veterinarians on different hairstyles but more permissive on appearance-related subjects.

## Introduction

In human medicine, the first impression is important for establishing a positive or negative relationship between the doctor and the patient^1^. Although dressing styles do not directly reflect the expertise and experience of doctors, human research indicates that it may influence the perceptions of the patients on doctors^2^. In general, patients prefer their doctors to be formally dressed because it makes them look more reliable, confident, and earnest^3,4^.

Compared with human medicine, only a few studies have investigated the relationship between veterinarian dressing styles and owner perceptions in veterinary medicine^3–8^. Most pet owners in an emergency setting had no preference regarding the dressing styles of veterinarians^5^. Similarly, another study that revealed that most owners considered it unnecessary for veterinarians to wear a white coat. In addition, wearing blue jeans, having colored hair, or having visible tattoos were not considered inappropriate for veterinarians^6^. However, two studies have revealed that dressing styles may influence owners’ trust in, confidence in, and comfort with a veterinarian^7,8^.

How pet owners and veterinarians regard appropriate dressing styles, hairstyle, and other exterior accessories for veterinarians could be different. In both human and veterinary medicine, the importance of tangibles, referring *to* physical facilities, appearance of personnel and equipment was shown likely to be overrated by the professionals, compared to the clients^9,10^. Also, one study conducted in the United Kingdom identified key attributes considered important in evaluating whether a veterinarian is perceived as “a good vet^11^”. Notably, a higher proportion of small animal veterinarians, compared to clients, regarded professional appearance as playing a significant role in defining a good veterinarian (*p* = 0.018). However, a subset of clients considered professional appearance “very important”, which represented a significantly higher proportion than that of small animal veterinarians (*p* = 0.019). No study that discerned the difference in the preference for attires between pet owners and veterinarians was found in the literature.

The aforementioned studies in veterinary medicine were all conducted in Western countries, and because of cultural differences, different results may be obtained in Asian countries. Thus, this study examined preferences for veterinarian dressing styles, hairstyles, and appearance-related subjects from both owner and veterinarian perspectives in Taiwan. Differences between male and female veterinarians were also analyzed.

## Results

### Respondents

A total of 303 responses were received (Table 1). Among the respondents, 211 (69.6%) and 92 (30.4%) were pet owners and veterinarians, respectively. About one-third of the respondents were females (*n* = 203; 67.0%). Compared with the age distribution of veterinarians, among which nearly 90% belonged to the group aged 21–30 years, pet owners were relatively equally distributed among age groups. Approximately two-thirds and one-third of pet owners went to the hospital to request a consultation for their dogs (*n* = 143; 67.8%) and cats (*n* = 62; 29.4%), respectively. Six owners (2.8%) preferred to visit female veterinarians, six (2.8%) preferred male veterinarians, and 199 (94.3%) did not have a sex preference. Owners had no sex preference for veterinarians (*p* < 0.001).

**Table 1:** Demographics of the respondents of a questionnaire asking the attitudes towards various dressing styles, hairstyles, and accessories of attending veterinarians from a teaching hospital in Taiwan.

Item	Category	All respondents	Owners	Veterinarians
Role	Pet owner	211 (69.6%)		
	Veterinarian	92 (30.4%)		
Gender	Female	203 (67.0%)	145 (68.7%)	58 (63.0%)
	Male	100 (33.0%)	66 (31.3%)	34 (37.0%)
Age group	20 years or less	5 (1.7%)	5 (2.4%)	0 (0%)
	21–30 years	135 (44.6%)	53 (25.1%)	82 (89.1%)
	31–40 years	69 (22.8%)	62 (29.4%)	7 (7.6%)
	41–50 years	45 (14.9%)	42 (19.9%)	3 (3.3%)
	51–60 years	25 (8.3%)	25 (11.8%)	0 (0%)
	61 years or more	24 (7.9%)	24 (11.4%)	0 (0%)
Preferred sex of veterinarians	No preference		199 (94.3%)	
	Female		6 (2.8%)	
	Male		6 (2.8%)	

### Dressing styles

**Fig. 1:**
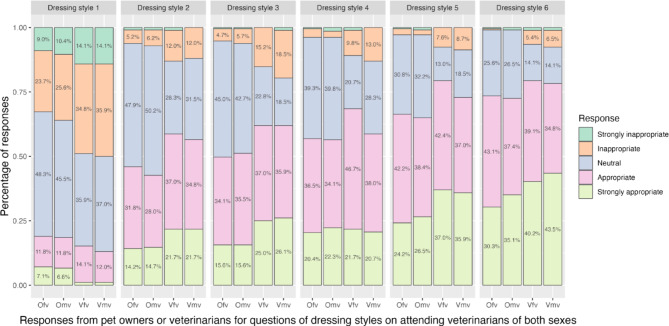
Responses to questions about the attitude toward different dressing style on female and male attending veterinarians, divided by respondent groups. Ofv: pet owners towards the question with female attending veterinarians in the dress; Omv: pet owners towards the question with male attending veterinarians in the dress; Vfv: veterinarians towards the question with female attending veterinarians in the dress; Vmv: veterinarians towards the question with male attending veterinarians in the dress.

According to the participants, the appropriateness of the six dressing styles increased from dressing style 1 to 6 (Fig. 1). Regardless of the respondents’ sex, dressing style 1 received much more negative responses than the rest of the styles. Female and male attending veterinarians in dressing style 1 were considered “strongly inappropriate” by 10.6% (*n* = 32) and 11.5% (*n* = 35) of the respondents, respectively, and “inappropriate” by 27.1% (*n* = 82) and 28.7% (*n* = 87) of the respondents, respectively. In contrast, most participants considered dressing styles 2–6 appropriate or neutral for veterinarians of both sexes, with only 2.6% (*n* = 8) to 10.2% (*n* = 31) of them considering these styles as either “strongly inappropriate” or “inappropriate.” Female and male attending veterinarians in dressing style 6 was considered the most appropriate among the participants.

The perception of the appropriateness of different dressing styles of attending veterinarians significantly differed between pet owners and veterinarians (Table 2). The difference was also observed for different dressing styles of attending veterinarians of different sexes in all styles except for the first one (*p* = 0.323). Generally, veterinarians appeared stricter on the inappropriateness of the dress code than pet owners (Fig. 1).

**Table 2:** The results for the comparison of the responses towards attending veterinarians and attending veterinarians of different sexes between pet owners and veterinarians from a teaching veterinary hospital in Taiwan.

Question	Adjusted *P* value for the comparison of the responses towards attending veterinarians between pet owners and veterinarians	Adjusted *P* value for the comparison of the responses towards attending veterinarians of different sexes between pet owners and veterinarians
Dressing style 1	0.025	0.323
Dressing style 2	< 0.001	0.010
Dressing style 3	< 0.001	< 0.001
Dressing style 4	< 0.001	0.005
Dressing style 5	< 0.001	0.005
Dressing style 6	< 0.001	0.003
Long hair	0.331	< 0.001
Medium hair	0.331	0.006
Short hair	1.000	0.926
Bald	0.283	0.003
Earring	< 0.001	< 0.001
Glasses	0.331	0.846
Name tag	1.000	1.000
Necklace	0.011	0.001
Ring	0.095	0.822
Sneaker	< 0.001	< 0.001
Stethoscope	0.028	0.323
Tattoo	0.025	0.192
Watch	1.000	1.000

### Hairstyles

**Fig. 2:**
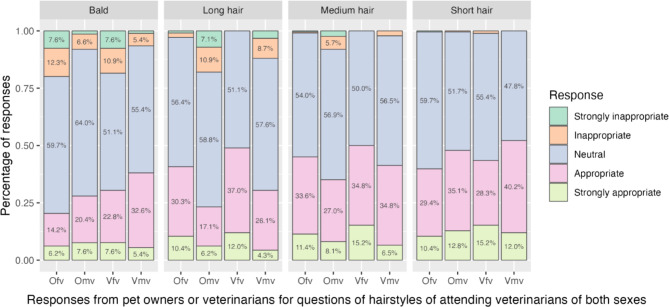
Responses to questions about the attitude toward different hairstyles of female and male attending veterinarians, divided by respondent groups. Ofv: pet owners towards the question for female attending veterinarians with the hairstyle; Omv: pet owners towards the question for male attending veterinarians with the hairstyle; Vfv: veterinarians towards the question for female attending veterinarians with the hairstyle; Vmv: veterinarians towards the question for male attending veterinarians with the hairstyle.

Most participants felt neutral or appropriate toward any hairstyle for female attending veterinarians, except for being bald, with about one-fifth (*n* = 59; 19.5%) of the participants considering it (strongly) inappropriate (Fig. 2). However, bald, long, and medium hairstyles were considered less appropriate by some participants for male attending veterinarians (7.6%, 16.1%, and 6.2%, respectively).

Regarding hairstyles, pet owners showed significantly stronger negative attitudes toward male attending veterinarians with long (*p* < 0.001) or medium (*p* = 0.006) hairstyles and bald female attending veterinarians than participating veterinarian (*p* = 0.003; Fig. 2; Table 2).

### Accessories

**Fig. 3:**
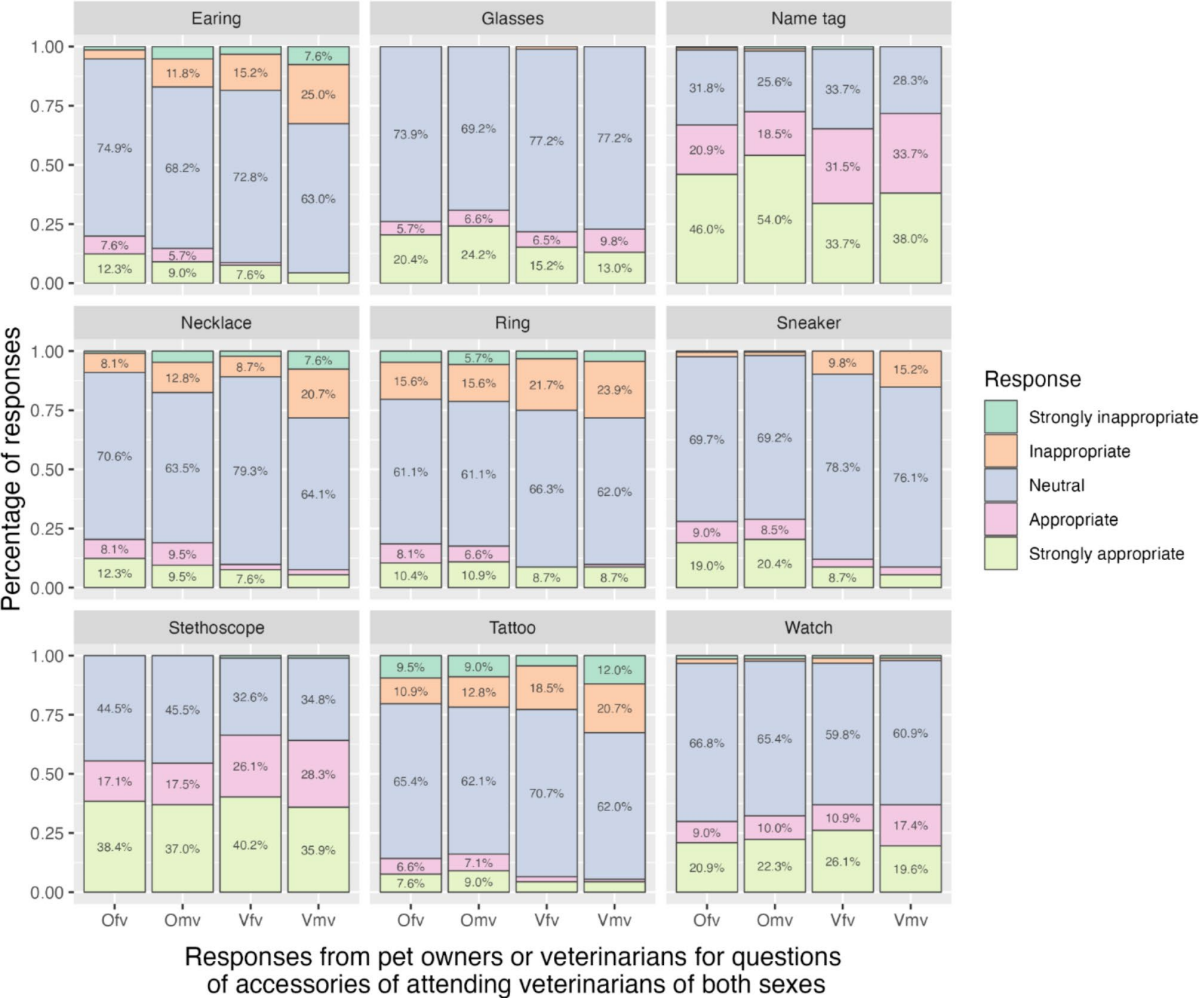
Responses to questions about the attitude toward the different accessories on female and male attending veterinarians, divided by respondent groups. OF: pet owners towards the question for female attending veterinarians with the accessory; Omv: pet owners towards the question for male attending veterinarians with the accessory; Vfv: veterinarians towards the question for female attending veterinarians with the accessory; Vmv: veterinarians towards the question for male attending veterinarians with the accessory.

The presence of a name tag (66.3% for female and 73.3% for male attending veterinarians) and a stethoscope (58.7% for female and 57.4% for male attending veterinarians) on veterinarians was considered (strongly) appropriate. Approximately one-fifth to one-fourth of the participants regarded wearing a ring and having tattoos as (strongly) inappropriate for attending veterinarians of both sexes. According to the participants, wearing earrings or necklaces was more than twice as unacceptable for male attending veterinarians than for female attending veterinarians. Few respondents considered wearing sneakers inappropriate for male and female attending veterinarians (6% and 4.6%). Almost no respondents thought wearing glasses, a name tag, a stethoscope, or a watch inappropriate (Fig. 3).

The appropriateness of wearing earrings (*p* < 0.001), a necklace (*p* = 0.011), and sneakers (*p* < 0.001) considered by pet owners and veterinarians was significantly different (Fig. 3; Table 2). Generally, veterinarians are stricter on the inappropriateness of the accessories than pet owners. Both veterinary and pet owner respondents strongly disapproved male attending veterinarians wearing earrings (*p* = < 0.001) or a necklace (*p* = 0.001), whereas only veterinary respondents appeared to disapprove more male attending veterinarians wearing sneakers (*p* < 0.001). The responses toward “stethoscope” (*p* = 0.028) and “tattoo” (*p* = 0.025) were significant between pet owners and veterinarians. Compared with pet owners, veterinarian respondents considered having tattoos more inappropriate and wearing a stethoscope more appropriate. However, the differences of attitudes were irrelevant to the sex of attending veterinarians.

### Perceptions of respondents of different sexes and age groups

When comparing perceptions of dressing styles, hairstyles, and accessories of attending veterinarians between male and female respondents, there was only one significant difference identified (Table 3). Male respondents expressed more disapproval than female respondents for dressing style 6 (scrub shirt + scrub trousers + coat; *p* = 0.012). Respondents of age 30 or less tended to express the preference of dressing styles and chose less “neutral” than those aging over 30 (Table 4). Additionally, respondents with age over 30 showed more disapproval towards bold hairstyle towards females (*p* = 0.047) and long (*p* = 0.002) and medium (*p* = 0.007) hairstyles towards males.

**Table 3:** Comparison of perceptions towards various dressing styles, hairstyles, and accessories of attending veterinarians between female and male respondents (i.e., pet owners and veterinarians) from a teaching hospital in Taiwan.

Category	Sex of respondents	Inappropriate	Neutral	Appropriate	Fisher’s exact test
Dressing style 1	Female	151 (37.2%)	182 (44.8%)	73 (18.0%)	0.459
	Male	85 (42.5%)	83 (41.5%)	32 (16.0%)	
Dressing style 2	Female	33 (8.1%)	171 (42.1%)	202 (49.8%)	0.607
	Male	18 (9.0%)	91 (45.5%)	91 (45.5%)	
Dressing style 3	Female	34 (8.4%)	152 (37.4%)	220 (54.2%)	0.543
	Male	22 (11.0%)	71 (35.5%)	107 (53.5%)	
Dressing style 4	Female	22 (5.4%)	149 (36.7%)	235 (57.9%)	0.270
	Male	16 (80.0%)	63 (31.5%)	121 (60.5%)	
Dressing style 5	Female	14 (3.5%)	104 (25.6%)	288 (71.0%)	0.132
	Male	13 (6.5%)	58 (29.0%)	129 (64.5%)	
Dressing style 6	Female	7 (1.7%)	83 (20.4%)	316 (77.8%)	0.012
	Male	10 (5.0%)	53 (26.5%)	137 (68.5%)	
Bald	Female	47 (11.6%)	247 (60.8%)	112 (27.6%)	0.700
	Male	35 (17.5%)	112 (56.0%)	53 (26.5%)	
Long hair	Female	37 (9.1%)	225 (55.4%)	144 (35.5%)	0.606
	Male	18 (9.0%)	118 (59.0%)	64 (32.0%)	
Medium hair	Female	12 (3.0%)	224 (55.2%)	170 (41.9%)	0.211
	Male	9 (4.5%)	108 (54.0%)	83 (41.5%)	
Short hair	Female	2 (0.5%)	212 (52.2%)	192 (47.3%)	0.139
	Male	1 (0.5%)	118 (59.0%)	81 (40.5%)	
Earing	Female	60 (14.8%)	288 (70.9%)	58 (14.3%)	0.788
	Male	34 (17.0%)	139 (69.5%)	27 (13.5%)	
Glasses	Female	1 (0.3%)	300 (73.9%)	105 (25.9%)	0.749
	Male	0 (0.0%)	144 (72.0%)	56 (28.0%)	
Name tag	Female	6 (1.5%)	116 (28.6%)	284 (70.0%)	0.763
	Male	2 (1.0%)	62 (31.0%)	136 (68.0%)	
Necklace	Female	62 (15.3%)	280 (69.0%)	64 (15.8%)	0.865
	Male	30 (15.0%)	135 (67.5%)	35 (17.5%)	
Ring	Female	96 (23.7%)	243 (59.9%)	67 (16.5%)	0.286
	Male	41 (20.5%)	133 (66.5%)	26 (13.0%)	
Sneaker	Female	18 (4.4%)	291 (71.7%)	97 (23.9%)	0.332
	Male	14 (7.0%)	144 (72.0%)	42 (21.0%)	
Stethoscope	Female	1 (0.3%)	171 (42.1%)	234 (57.6%)	0.785
	Male	1 (0.5%)	81 (40.5%)	118 (59.0%)	
Tattoo	Female	90 (22.2%)	263 (64.8%)	53 (13.1%)	0.636
	Male	50 (25.0%)	128 (64.0%)	22 (11.0%)	
Watch	Female	9 (2.2%)	259 (63.8%)	138 (34.0%)	0.343
	Male	8 (4.0%)	131 (65.5%)	61 (30.5%)	

**Table 4:** Comparison of perceptions towards various dressing styles, hairstyles, and accessories of attending veterinarians between respondents of different age groups from a teaching hospital in Taiwan.

Category	Sex of respondents	Inappropriate	Neutral	Appropriate	Fisher’s exact test
Dressing style 1	Age 30 and less	117 (41.8%)	109 (38.9%)	54 (19.3%)	0.082
	Over age 30	119 (36.5%)	156 (47.9%)	51 (15.6%)	
Dressing style 2	Age 30 and less	23 (8.2%)	99 (35.4%)	158 (56.4%)	0.001
	Over age 30	28 (8.6%)	163 (50.0%)	135 (41.4%)	
Dressing style 3	Age 30 and less	38 (13.6%)	70 (25.0%)	172 (61.4%)	> 0.001
	Over age 30	18 (5.5%)	153 (46.9%)	155 (47.5%)	
Dressing style 4	Age 30 and less	25 (8.9%)	76 (27.1%)	179 (63.9%)	> 0.001
	Over age 30	13 (4.0%)	136 (41.7%)	177 (54.3%)	
Dressing style 5	Age 30 and less	19 (6.8%)	49 (17.5%)	212 (75.7%)	> 0.001
	Over age 30	8 (2.5%)	113 (34.7%)	205 (62.9%)	
Dressing style 6	Age 30 and less	15 (5.4%)	53 (18.9%)	212 (75.7%)	> 0.001
	Over age 30	2 (0.6%)	83 (25.5%)	241 (73.9%)	
Bald	Age 30 and less	26 (9.3%)	175 (62.5%)	79 (28.2%)	> 0.001
	Over age 30	56 (17.2%)	184 (56.4%)	86 (26.4%)	
Long hair	Age 30 and less	12 (4.3%)	171 (61.1%)	97 (34.6%)	0.002
	Over age 30	43 (13.2%)	172 (52.8%)	111 (34.0%)	
Medium hair	Age 30 and less	3 (1.1%)	167 (59.6%)	110 (39.3%)	0.453
	Over age 30	18 (5.5%)	165 (50.6%)	143 (43.9%)	
Short hair	Age 30 and less	1 (0.4%)	160 (57.1%)	119 (42.5%)	0.017
	Over age 30	2 (0.6%)	170 (52.1%)	154 (47.2%)	
Earing	Age 30 and less	53 (18.9%)	195 (69.6%)	32 (11.4%)	0.040
	Over age 30	41 (12.6%)	232 (71.2%)	53 (16.3%)	
Glasses	Age 30 and less	0 (0.0%)	218 (77.9%)	62 (22.1%)	0.024
	Over age 30	1 (0.3%)	226 (69.3%)	99 (30.4%)	
Name tag	Age 30 and less	1 (0.4%)	89 (31.8%)	190 (67.9%)	0.088
	Over age 30	7 (2.1%)	89 (27.3%)	230 (70.6%)	
Necklace	Age 30 and less	46 (16.4%)	194 (69.3%)	40 (14.3%)	0.385
	Over age 30	46 (14.1%)	221 (67.8%)	59 (18.1%)	
Ring	Age 30 and less	71 (25.4%)	172 (61.4%)	37 (13.2%)	0.195
	Over age 30	66 (20.2%)	204 (62.6%)	56 (17.2%)	
Sneaker	Age 30 and less	18 (6.4%)	214 (76.4%)	48 (17.1%)	0.005
	Over age 30	14 (4.3%)	221 (67.8%)	91 (27.9%)	
Stethoscope	Age 30 and less	2 (0.7%)	107 (38.2%)	171 (61.1%)	0.086
	Over age 30	0 (0.0%)	145 (44.5%)	181 (55.5%)	
Tattoo	Age 30 and less	55 (19.6%)	192 (68.6%)	33 (11.8%)	0.128
	Over age 30	85 (26.1%)	199 (61.0%)	42 (12.9%)	
Watch	Age 30 and less	5 (1.8%)	183 (65.4%)	92 (32.9%)	0.397
	Over age 30	12 (3.7%)	207 (63.5%)	107 (32.8%)	

## Discussion

Veterinarian dressing styles may play an important role in the first impression of pet owners^1^, and the current study identified important gaps in the perception of dressing styles between pet owners and veterinarians in Taiwan. Different dressing styles, hairstyles, and accessories for male and female veterinarians were perceived with varying degrees of appropriateness. Veterinary practitioners can mind our attire according to the results of the current study to form a more reliable relationship between owners and veterinarians.

A previous study in veterinary medicine indicated that owners feel greater trust in and comfort with female veterinarians than male veterinarians^7^. Owners were also more likely to share medical and social information regarding their pet to a female veterinarian^12^. In addition, human patients appear less intimidated by female doctors and expect female doctors to be more interested in their emotions^13^. However, no sex preference for the veterinarian was found in the current study.

Most of our respondents considered “shirt + trousers” and “scrub shirt + scrub trousers + coat” the most inappropriate and appropriate dressing styles for veterinarians, respectively. Our results were similar to those of studies in veterinary medicine. Coe et al. concluded that the most preferred dressing style is wearing a surgical scrub, followed by white coat, whereas wearing casual shirt and jeans is the most disliked dressing style^7^. Bentley et al. stated that owners perceived veterinarians wearing white coats and surgical scrubs over casual business attire as more competent and have higher comfort levels^8^. According to Sugerman-McGriffin et al., surgical and clinical dressing styles were more preferred by owners over business and casual dressing styles^5^. Robb et al. reported that 70% of owners thought that veterinarians did not have to wear white coats; however, most respondents still preferred veterinarians wearing a white coat^6^. Therefore, if veterinary practitioners want to leave a positive first impression on the owners, wearing either a white coat or surgical scrubs is appropriate for veterinarians in both Western and Eastern countries.

To the best of our knowledge, this is the first study to explore pet owners’ perceptions of veterinarians’ hairstyles. For female attending veterinarians, almost all hairstyles were considered appropriate or neutral, except that about one-fifth of owners found a bald hairstyle inappropriate. Traditional Asian beauty standards often emphasize long, flowing hair as a symbol of femininity^14^. This may explain the negative perceptions of baldness in women, as it deviates from the cultural norm. Similarly, while 99.6% of the owners regarded short hair being neutral or appropriate, 17.9% of the owners considered long hairstyles inappropriate. These findings are consistent with studies from the United States^15^ and Korea^16^. These studies found that short hairstyles are linked to professionalism. In contrast, long hairstyles may negatively impact perceptions of competence and leadership potential, affecting career opportunities. Generation was also found to be a factor associated with the perceptions towards hairstyles, as older generation seems to associate professionalism with traditional and conservative appearances more, including hairstyles^17^. In the current study, respondents over the age of 30 expressed greater disapproval of non-culturally and sexually conforming hairstyles compared to younger respondents. In general, pet owners seemed less comfortable with diverse hairstyles than veterinarian respondents. Veterinarians can use these findings to inform their hairstyle choices. However, promoting diversity, equity, and inclusion at the facility level may help challenge false associations between hairstyles and competence.

Wearing accessories such as earrings, necklaces, rings, or tattoos was generally perceived as inappropriate, with male attending veterinarians receiving higher rates of disapproval compared to female attending veterinarians. Interestingly, veterinarians were more critical of these accessories than pet owners. These findings suggest that such accessories are not viewed as enhancing the professional image of veterinarians. On the contrary, wearing glasses, a name tag, a stethoscope, or a watch was almost without negative impressions from the results of both owners and veterinarians. An interesting finding in the results of wearing sneakers was noted: 15.2% and 9.8% of the veterinarians think that wearing sneakers was inappropriate for male and female attending veterinarians, respectively; however, only 1.9% and 2.4% of the owners think it was inappropriate. Therefore, wearing sneakers would not negatively affect the professional image of attending veterinarians for most clients in Taiwan, and veterinarians may adapt the point-of-view accordingly.

This study has some limitations. First, this study included twice as many female respondents as male respondents. We are not sure if this population can reflect the true opinion of the owners. Second, the results may vary in different settings, such as emergency or specialty settings. This study was conducted in a national teaching hospital; therefore, it may not reflect the true condition in the first-line clinics. Also, some demographics characteristics such as profession, reason for going to the veterinarian, household income, and social status might be relevant to the preference for and the attitude towards the dressing of veterinarians^7,8^. Future study may further investigate. Lastly, the respondents may be concerned about the consequences of giving truthful answers to some sensitive questions, which is called “thread of disclosure”^18^. To prevent this condition, a QR code was provided for owners to answer this questionnaire without recording personal information. We believe that this method can present a more reliable result.

In conclusion, both pet owners and veterinarians showed a preference for veterinarians’ dressing style, and how pet owners regard appropriate veterinarians’ dressing style may influence their first impression of the veterinarian. Wearing a white coat or surgical scrubs is appropriate for veterinarians. Any hairstyle, except for a bald one, was regarded appropriate for female veterinarians, and male veterinarians with short hair can give a more appropriate impression to the owners. Wearing glasses, a name tag, a stethoscope, a watch, and sneakers tends to leave a neutral or appropriate impression on the owners. Wearing an earring, necklace, ring, or tattoo was considered much more inappropriate by some owners and should be avoided, particularly for male veterinarians. Overall, the owners were stricter than the veterinarians regarding different hairstyles but more permissive on appearance-related subjects.

## Methods

### Study population and design

The study was conducted at the National Taiwan University Veterinary Hospital, and an anonymous questionnaire was posted online using Google Forms. Pet owners visiting the hospital and veterinarians working there were invited to participate in this study. This study was reviewed for ethics evaluation and was approved for clinical research by the Committee of National Taiwan University Veterinary Hospital (No. NTUVH111001). All methods were performed in accordance with the relevant guidelines and regulations. All participants received a link to provide consent, completed the questionnaires and provided informed consent.

### Questionnaire design and validity

**Fig. 4:**
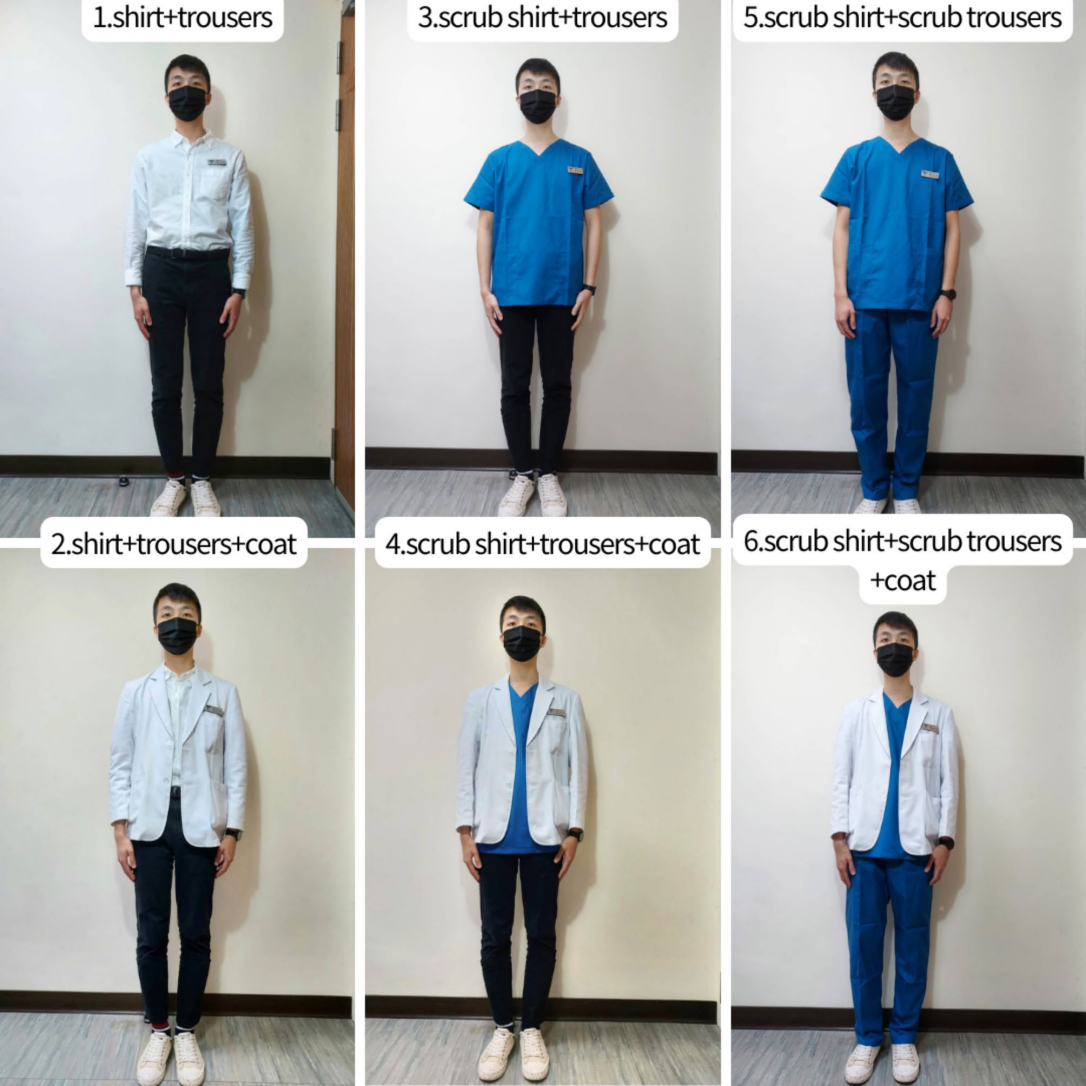
Photographs of different attires to male veterinarians.

**Fig. 5:**
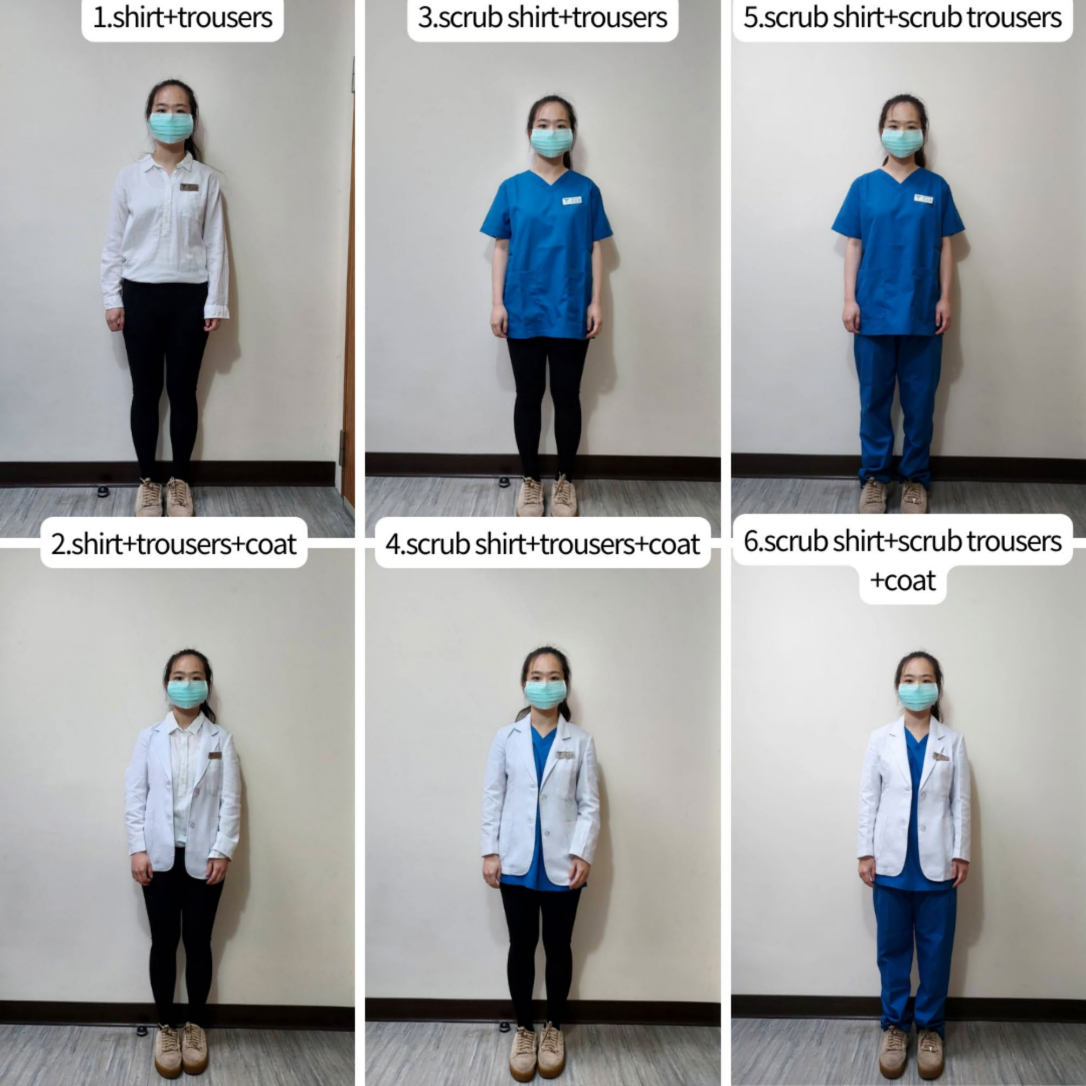
Photographs of different attires to female veterinarians.

The questionnaire contained three modules. The first module focused on the demographics of the participants, asking for information about their role (i.e., veterinarians or pet owners), sex, age, type of pet, and sex preference for veterinarians. The second and third modules investigated the attitudes of the participants toward different aspects of the appearance of attending veterinarians of each sex, and participants were asked to rate their opinions on the suitability of the dressing style according to a 5-point Likert scale (1 = strongly inappropriate, 2 = inappropriate, 3 = neutral, 4 = appropriate, and 5 = strongly appropriate). In the second module, photos of six clothing styles of both male and female attending veterinarians were included, namely, shirt + trousers, shirt + trousers + coat, scrub shirt + trousers, scrub shirt + trousers + coat, scrub shirt + scrub trousers, and scrub shirt + scrub trousers + coat (Figs. 4 and 5). The third module further asked about whether different hairstyles (bald, short, medium, and long hair hairstyles), carrying a stethoscope, wearing a name tag, wearing glasses, wearing a watch, wearing a ring, wearing an earring, wearing a necklace, having a tattoo, and wearing sneakers were appropriate in both male and female attending veterinarians.

Face validity, which is the degree to which the questionnaire appears to assess the desired qualities, was established through informal discussions with veterinarians and technicians at the National Taiwan University Veterinary Hospital and their feedback^19^. The survey was officially open to the participants after being tested with 10 veterinarians and 10 pet owners and revised according to their suggestions.

### Study implementation

From February 1, 2022, to July 31, 2022, the QR code of the online questionnaire was posted in the waiting area of the surgical, internal medicine, and exotic animal sections at the National Taiwan University Veterinary Hospital. Potential participants, including pet owners and veterinarians working at the teaching hospital were encouraged to scan the QR code linked to the questionnaire.

### Statistical analysis

Data cleaning and management were conducted using Microsoft Excel 2016 and R version x64 4.2.2 with R packages “midverse” and “rio” in RStudio interface^20^. Descriptive statistics that summarize the collected data were also performed in R with “tidyverse,” “janitor,” “ggpubr,” and “strex.” Fisher’s exact test was conducted in R to examine whether pet owners and veterinarians had different perceptions of each question for attending veterinarians of both sexes combined, as well as whether pet owners and veterinarians had different perceptions toward female and male attending veterinarians for the same question. Fisher’s exact test was also used to compare the perceptions between female and male respondents, as well as participants of ≤ age of 30 and over age of 30. The tests used recategorized outcomes with “strongly inappropriate” and “inappropriate” as “inappropriate,” “neutral” as “neutral,’ and “appropriate” and “strongly appropriate” as “appropriate.” With multiple testing, the P values were adjusted at the false discovery rate^20^. Any *p* < 0.05 was considered significant.

## Data Availability

The data presented in this study are available on request from the corresponding author.
